# Neural crest streaming as an emergent property of tissue interactions during morphogenesis

**DOI:** 10.1371/journal.pcbi.1007002

**Published:** 2019-04-22

**Authors:** András Szabó, Eric Theveneau, Melissa Turan, Roberto Mayor

**Affiliations:** Research Department of Cell and Developmental Biology, University College London, London, United Kingdom; Oxford, UNITED KINGDOM

## Abstract

A fundamental question in embryo morphogenesis is how a complex pattern is established in seemingly uniform tissues. During vertebrate development, neural crest cells differentiate as a continuous mass of tissue along the neural tube and subsequently split into spatially distinct migratory streams to invade the rest of the embryo. How these streams are established is not well understood. Inhibitory signals surrounding the migratory streams led to the idea that position and size of streams are determined by a pre-pattern of such signals. While clear evidence for a pre-pattern in the cranial region is still lacking, all computational models of neural crest migration published so far have assumed a pre-pattern of negative signals that channel the neural crest into streams. Here we test the hypothesis that instead of following a pre-existing pattern, the cranial neural crest creates their own migratory pathway by interacting with the surrounding tissue. By combining theoretical modeling with experimentation, we show that streams emerge from the interaction of the hindbrain neural crest and the neighboring epibranchial placodal tissues, without the need for a pre-existing guidance cue. Our model suggests that the initial collective neural crest invasion is based on short-range repulsion and asymmetric attraction between neighboring tissues. The model provides a coherent explanation for the formation of cranial neural crest streams in concert with previously reported findings and our new in vivo observations. Our results point to a general mechanism of inducing collective invasion patterns.

## Author summary

A central question in morphogenesis is how complexity arises from unpatterned tissues. One crucial event in vertebrate development is the migration of neural crest cells into stereotypic streams. Cranial neural crest cells start their migration as a single tissue mass but invade their environment and migrate in distinct streams. While this stream migration is crucial for correct head development and is relatively well studied, it is unclear how the initial stream patterns are formed. Surrounding tissues lack a clear organization prior to neural crest migration, making the existence of a guiding pre-pattern unlikely. In this study we address the question of how the initial neural crest streams are formed by combining theoretical modeling with experimentation and show that neural crest streams emerge from dynamic interactions with neighboring tissues.

## Introduction

Shape often plays an essential role for organ function. Therefore, understanding the process of shape acquisition, called morphogenesis, is crucial to understanding developmental processes and how to prevent their breakdown in pathologies. Studies over the last century identified a handful of universal modules controlling tissue morphogenesis, such as the spreading and thinning of epithelial sheets (epiboly) or convergent extension [1]. Most studies aim to understand morphogenesis without the need to consider environmental effects [2,3] despite the fact that developing tissues interact dynamically with their embryonic environment.

**Fig 1:**
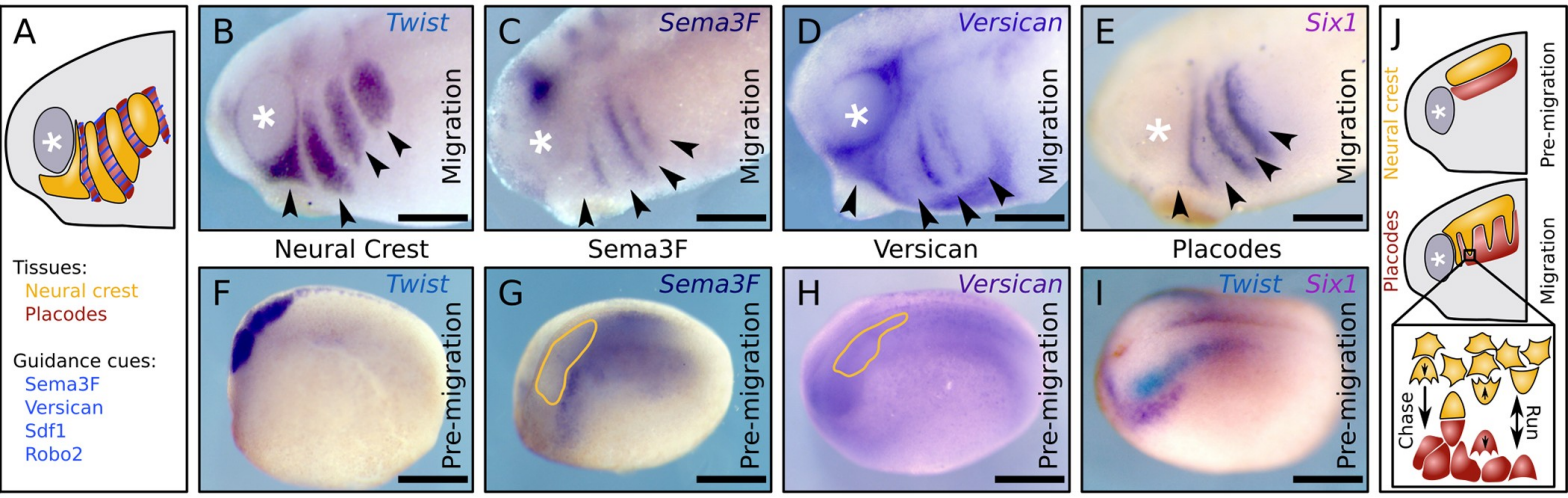
The neural crest and its surroundings before and during migration. (A) Cranial neural crest (NC) migrates in streams that are flanked by a variety of essential guidance cues corresponding with the epibranchial placodes. (B) Lateral view of Xenopus laevis embryo showing migrating NC by in situ hybridization (ISH) against Twist. (C-E) ISH showing expression of NC inhibitors Semaphorin 3F (C) and Versican (D), and the coinciding placodal cell pattern (E, Six1) during NC migration. (F-I) ISH showing pre-migratory NC, inhibitor expressions, and placode distribution before NC migration. Yellow outline on (G,H) indicate position of NC; (I) shows NC (Twist, blue) and placodes (Six1, purple) by double-ISH and embryo is shown in a slightly tilted dorso-lateral view. Twist adjacent to Six1 marks cranial NC. (J) Illustration of known NC-placode cell interactions and the hypothesis that this interaction leads to emergence of the NC stream pattern. Arrowheads on (B-E) indicate NC streams, ‘*’ marks the eye/ optic vesicle; scalebars: 300μm.

A striking example for the importance of environmental interactions during morphogenesis is the migration of the neural crest (NC). NC cells, an embryonic cell population whose migratory behavior has been likened to cancer invasion, are formed along the neural tube in the ectoderm and undergo an epithelial-to-mesenchymal transition (EMT) to form a single bulk pre-migratory NC tissue [4]. Subsequently, the NC cells are gathered into characteristic streams (Fig 1A and 1B) in which they migrate large distances collectively to contribute to a number of organs and give rise to a multitude of cell types [4]. Proper NC migration relies on environmental cues such as Semaphorin-3F (mouse) [5], or Versican (frogs) [6], Sdf1 (frog and fish) [7], Robo2 (chick) [8] (Fig 1A), as well as combinations of Eph-Ephrins (frogs) [9].

In the head, inhibitory cues such as Semaphorin-3F, Versican, and others are expressed in the epibranchial placodal cells surrounding the migratory streams originating from the hindbrain in the frog *Xenopus laevis* (Fig 1A, 1C–1E). In addition, EphA4, EphB1, and ephrin-B2 are expressed in the NC and matching mesoderm in *Xenopus* [9]. Blocking the function of these inhibitors impairs the correct formation of the NC streams in mice and frogs [5,6,9], suggesting that NC streams are shaped by a pre-existing environmental pattern of inhibitors that funnels the NC into correct migratory pathways as suggested for mice [10]. However, prior to migration the inhibitors are not patterned along the presumptive NC streams in the epibranchial region as they are expressed as a continuous band adjacent to the NC (Fig 1F–1I). Therefore, how the initial formation of the NC streams is established remains unknown.

Although several computational studies have been published on NC migration previously [6,11–16], these mostly focused on stream directionality and cell-cell coordination during collective migration. Therefore, the question of how NC streams are generated has remained unexplored.

Here we investigate the idea that initial establishment of NC migratory streams results from the dynamic interactions of the NC and placodal tissues (Fig 1J). Placodal cells at this stage of development are motile and form a loose epithelial tissue in frogs and fish [7]. NC and placodes in these species are engaged in repulsion called contact inhibition of locomotion (CIL), whereby placodal and NC cells re-polarize and move away from one another after coming into contact as described for frogs and fish [17]. In addition, placodes attract adjacent NC cells through Sdf1 chemotaxis. The combination of these activities leads to a persistent directional migration of NC cells in a process named ‘chase and run’ which is essential for NC migration in frogs and fish [7]. We tested in silico and in vivo whether this chase and run interaction could lead to the initial emergence of NC streams, challenging the need for a pre-pattern to funnel the NC into streams.

## Results

### Cell-based simulations of neural crest—Placode interactions predict emergent streams

**Fig 2:**
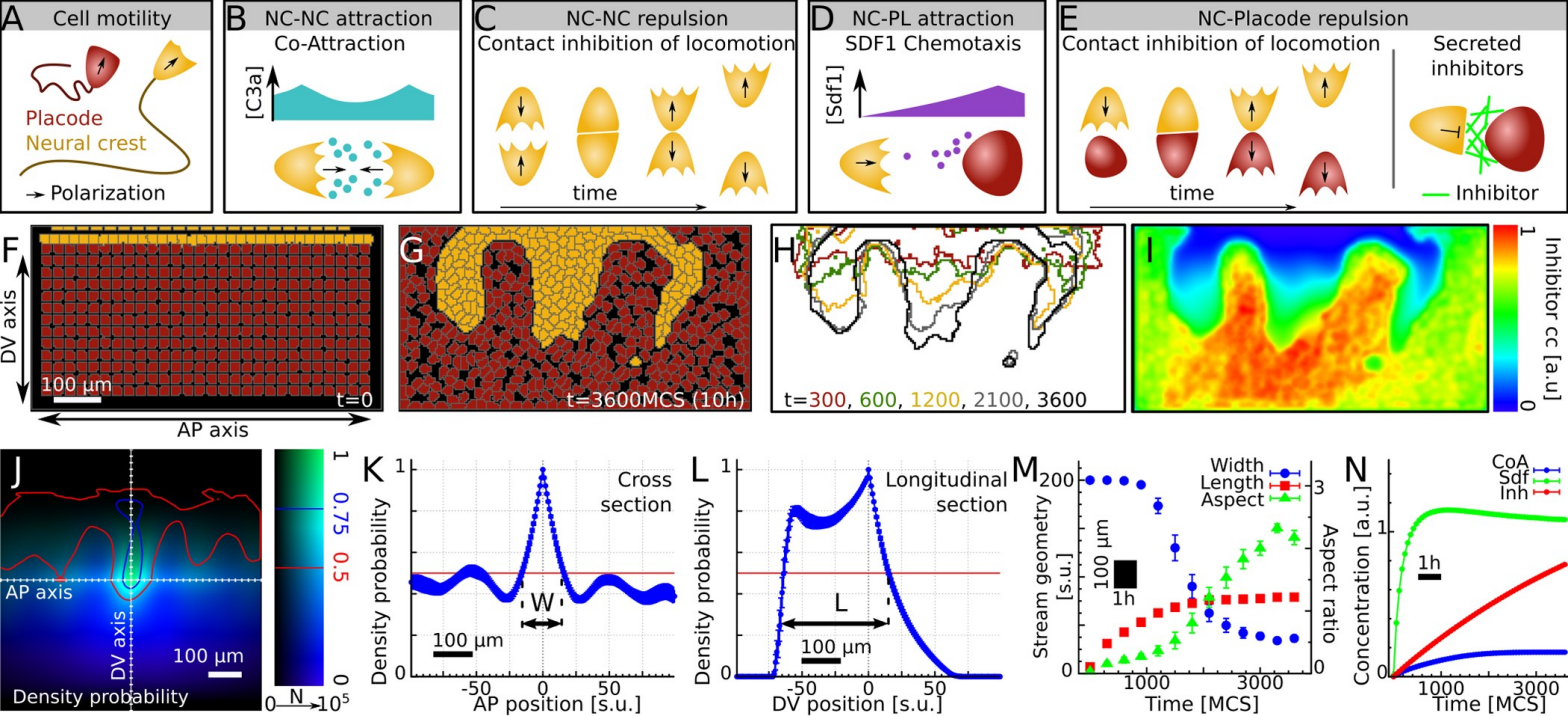
Cell-based model of neural crest—placode interactions. (A-E) Illustration of cellular behaviors in the model: polarized cell motility (A), co-attraction (CoA, B) and contact inhibition of locomotion (CIL, C) between NC-NC cells, and chase and run behavior (D-E) based on Sdf1 chemotaxis (D) and repulsion (E) between NC-placode cells. (F-G) Example simulation of the model, showing initial (E) and final (F) cell configurations with NC (orange) and placodal (red) cells. AP and DV axes indicate axes analogous to anterior-posterior and dorso-ventral directions in embryos. (H) Outline of the simulated NC population at *t* = 300,600,1200,2100, and 3600 MCS. (I) Inhibitor concentration levels at the end of the simulation mimicking the pattern of inhibitors in vivo. (J) NC cell density probability function related to pair-correlation of cells. Color indicates $\rho (\vec{r})$ value, brightness shows number of samples (*N*) at position $\vec{r}$ in summation (Eq 1); averaged from n = 20 independent simulation repeats. (K-L) Sections of $\rho (\vec{r})$ function shown in (J) along the AP (K) and DV (L) axes. *W* and *L* indicate the characteristic width and length. (M) Time evolution of *W*, *L*, and the aspect ratio *L*/*W*. (N) Average concentration levels in the simulation area. Error bars indicate SEM; n = 20 simulations; a.u.: arbitrary unit; s.u.: simulation unit of one lattice site, the minimal simulated distance; MCS: Monte Carlo time step.

To test whether cellular interactions can lead to formation of streams, we extended our previous model focusing solely of NC migration [6] by introducing placodal cells as detailed in the Materials and Methods section. Assumptions of the model are based on previously described behavior of *Xenopus* cranial NC. Briefly, two cell types (NC and placodes) are included in a cellular Potts model. Cells are self-propelled along their direction of polarization with higher (NC) or lower (placodes) motility persistence (Fig 2A) resulting in higher cell speeds for NC than placodal cells. Note that in vivo NC and placodal cells use an underlying fibronectin matrix as a migratory substrate. It is known that NC cells secrete a diffusible molecule, complement component C3a, which works as an attractant for the NC themselves in a process named ‘co-attraction’ (CoA) [11]. In the model CoA of NC is implemented through secretion of a NC chemoattractant (Fig 2B). It has been shown in frog and fish that when two NC cells come into contact they undergo a process termed contact inhibition of locomotion (CIL) [17,18]. In the model, the polarization vector of contacting cells is biased away from the contact, eventually leading to the separation of the two cells (Fig 2C). CIL and CoA together constitute repulsion upon contact and attraction at a distance between NC cells. Similarly, it has been demonstrated in frog that NC cells are attracted to Sdf1 secreted by the placodal cells [17,18], which is implemented in the model (Fig 2D). It is also known that NC cells and placodes in frogs exhibit a short-range repulsion [7]. This is implemented as CIL between these cells (Fig 2E). Additionally, based on the expression patterns of inhibitor molecules surrounding the NC streams (Fig 1C and 1D), we assume that placodes secrete molecules that act to confine the migration of NC cells (Fig 2E). For simplicity, we model all inhibitory molecules as a single combined repulsive signal with an extremely short diffusion range (half-cell diameter) describing pericellular deposition of the signal. Attraction via Sdf1 and repulsion at a shorter range together result in a chase-and-run behavior between the NC and the placodes, whereby NC approach the placodes due to Sdf1 chemotaxis (‘chase’ phase), establish contact with the placodes, and are repelled from the contact site causing both NC and placodes to move away from each other (‘run’ phase) [7]. Placodal cells neither attract each other, nor do they exhibit CIL behavior [7]. Table 1 summarizes the cell-cell interactions in the NC-placode model.

**Table 1:** Summary of main interactions between cell types in the model.

	NC cell	Placodal cell
NC cell	Long range attraction (Co-Attraction) Short range repulsion (CIL)	Long range attraction (Sdf1 chemotaxis) Short range repulsion (CIL)
Placodal cell	Long range attraction (Sdf1 chemotaxis) Short range repulsion (CIL)	

At the start of simulations, placodal cells are initialized in 13 rows and NC cells in 2 rows in a rectangular area with closed boundary conditions (Fig 2F). To mimic the progressive production of NC cells by EMT, the top region of the simulation area is monitored for empty space. Whenever a cell-free space sufficient to accommodate half a cell is detected, a new NC cell is introduced into the simulation at that space.

Simulations of the model show that NC cells invade the placodal region in distinct streams (Fig 2G and 2H, S1 Movie) without the need for any pre-pattern. While initially all NC cells facing the placodes experience the same micro-environment, parts of the interface later become specified as regions of invasion. Together with the NC streams, a pattern of the inhibitor also emerges in the simulations (Fig 2I, S2 Movie) matching the NC stream pattern similarly as observed in vivo (Fig 1C and 1D).

Streams in the model are characterized using a NC density probability function, similar to a pair correlation function of cell positions, defined as:

$$\rho (\vec{r})=\langle \eta (\vec{r}+\vec{R})\rangle_{\vec{R}:\eta (\vec{R})=1}$$ (1)

where $\eta (\vec{R})=1$ only if position $\vec{R}$ is occupied by a NC cell, otherwise 0. The symbol $\langle \ldots \rangle_{\vec{R}:\eta (\vec{R})=1}$ denotes average over all possible $\vec{R}$ positions where $\eta (\vec{R})=1$. Therefore, $\rho (\vec{r})$ approximates the probability of finding two NC cells at position $\vec{r}$ relative to each other. In configurations with streams (Fig 2G) this function outlines the local neighborhood of an average NC cell reminiscent of a stream (Fig 2J). Sections along the axes of $\rho (\vec{r})$ show average stream profiles (Fig 2K and 2L). A characteristic width, *W* = 36 ± 3 lattice sites (±SEM), and length, *L* = 79 ± 5 lattice sites (±SEM), are established as the distance of the two loci where the profiles first fall below 0.5, denoting 50% probability of NC cell presence. Time evolution of these measures and the aspect ratio *L*/*W* in the simulations shows that streams progressively reduce their width and increase in length up to a point after which the streams stabilize (Fig 2M); note that the streams do not break into segregated clusters, even if the simulations are run for a longer time. This is accompanied by the saturation of the chemoattractant Sdf1, while inhibitor levels are still increasing (Fig 2N), leading to stabilization of the emergent geometry. This dynamic formation and evolution of streams in the model is similar to the migration of NC observed in vivo in frogs and fish [7,11].

In summary, our model predicts that NC streams may emerge from dynamic interactions between NC and placodal cells in the absence of an underlying guidance pattern.

### Ectopic neural crest forms streams and affects surrounding inhibitory signal expression

**Fig 3:**
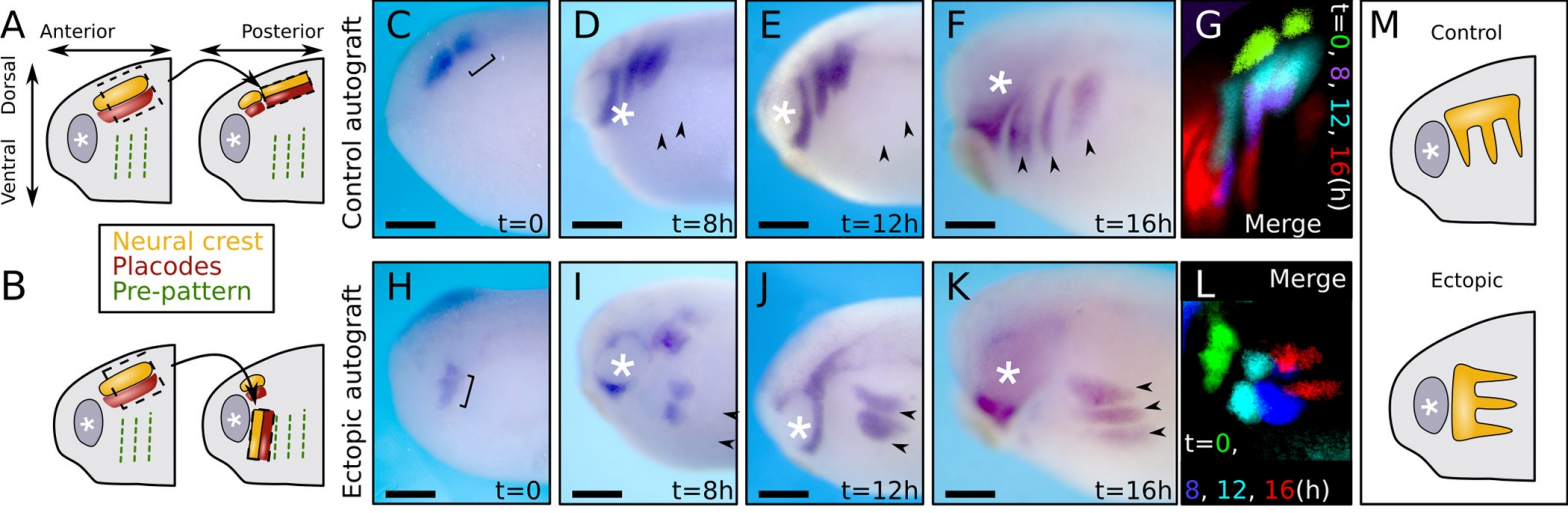
Ectopic neural crest is able to invade normally NC-free territories and form streams. (A-B) Illustration of autograft experiments showing the control (A) and ectopic-rotated (B) graft arrangements. (C-F) Control experiment after graft healing (C, t = 0) and t = 8h (D), 12h (E), and 16h (F) later. (G) Pseudo-colored overlay of control grafted embryos shown on (C-F). (H-K) Ectopic autograft after healing (H, t = 0) and t = 8h (I), 12h (J), and 16h (K) later. (L) Pseudo-colored overlay of ectopic grafts shown on (H-K). (M) Illustration of stream formation observed in control and ectopic graft experiments. ISH against Twist; arrowheads indicate streams in the grafted locations; ‘*’: eye / optic vesicle; braces indicate initial graft; scalebars: 300μm.

We tested the prediction of our model that NC streams emerge independently from an underlying guiding pattern as suggested by the prevalent view; that is: a set of pre-existing lines that guide the NC into streams. To test this experimentally, we surgically removed the NC and its neighboring placodal population from pre-migratory stage *Xenopus laevis* embryos (stage 16–18 N&F [19]) and grafted them in the same embryo in either their normal position and orientation as control (Fig 3A) or at an angle of 90° (Fig 3B) within the pharyngeal region. This was done by opening the epidermis to retrieve the NC and placodes. The epidermis and the mesoderm have a relatively low adhesion to the NC and placode tissues, allowing to easily separate them. After re-positioning the NC and placodal tissues the epidermis is re-closed. Importantly, the mesoderm and overlying epidermis were neither transferred nor removed during the surgery. At these stages the prospective placodes form a continuous, approximately 5 cell thick band adjacent to the NC, called preplacodal region; this region lacks any obvious pattern in vertebrates [20]. The position of the NC was assessed by in situ hybridization (ISH) against the NC marker *Twist* at 0, 8, 12, and 16 hours after the graft healed. If the migration of NC into streams is dependent on a pre-pattern present in the embryo, the rotated NC should not form normal streams as they will not reach this hypothetical pre-pattering (Fig 3B).

The control NC graft migrated to form streams in the normal orientation and position (Fig 3C–3F), whereas the rotated NC grafts did not follow the path of normal NC migration, but still formed distinct streams that migrated according to their rotated orientation perpendicularly with respect to the control streams (Fig 3H–3M). These results show that NC stream formation can occur irrespective of the orientation of the under- or overlying tissues and support our model whereby the initial formation of NC streams is not governed by a pre-pattern.

In the following we test the proposed model by exploring its behavior under perturbed conditions in order to support our main results presented above.

### Number of streams is determined by migratory region width

**Fig 4:**
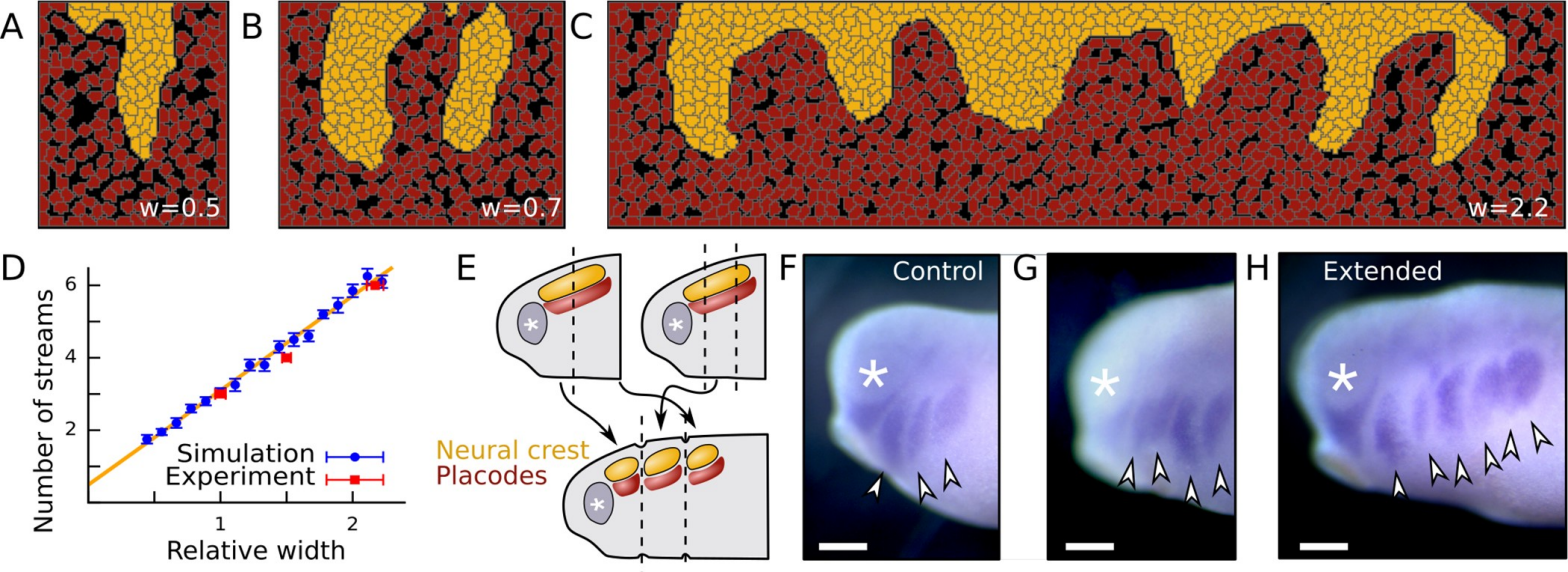
Number of streams is determined by the width of migratory region. (A-C) Final cell configurations in simulations with varying widths (w) relative to the normal simulation width shown in Fig 3. (D) Number of streams (n) versus relative system width (w) in simulations and in vivo. Error bars: SEM; n = 20 simulations; n = 5 embryos; yellow line indicates linear fit: n(w) = 2.61*w+0.49. (E) Illustration of experiments for perturbing the width of the cranial NC migratory region. (F-H) NC streams revealed by ISH in control (F) and in extended (G, H) cranial NC regions. Lateral view; *, eye; arrowheads, streams; scalebars: 300μm.

To further explore how the closed boundary condition along the AP axis in our model affects stream formation, a series of simulations were performed using a range of system widths. In these simulations the initial number of cell rows was kept constant therefore keeping the same linear cell densities. We observed stream formation irrespective of the system width (Fig 4A–4C) and found a direct relationship between the number of streams and system width (Fig 4D).

The in silico results were compared with in vivo behavior by performing a series of graft experiments in which the cranial region was extended by grafting segments of NC and placodes, containing the equivalent region from another embryo (Fig 4E). Similar to our in silico results and control embryos, the extended segments were able to give rise to NC streams (Fig 4F–4H). We found a similar relationship between the extension ratio and the number of streams in vivo as predicted by the simulations (Fig 4D).

Together these results indicate that the number of migratory streams is determined by the space available for the NC along the AP axis. Note that these results do not test the lack of pre-pattern and in themselves could be compatible with a pre-pattern driven stream formation. In vivo, these boundaries are most likely the forming eye from the anterior and Semaphorin-3A at the posterior boundary of the epibranchial region in frogs [21].

### Long-range attraction and short-range repulsion are required for stream emergence

**Fig 5:**
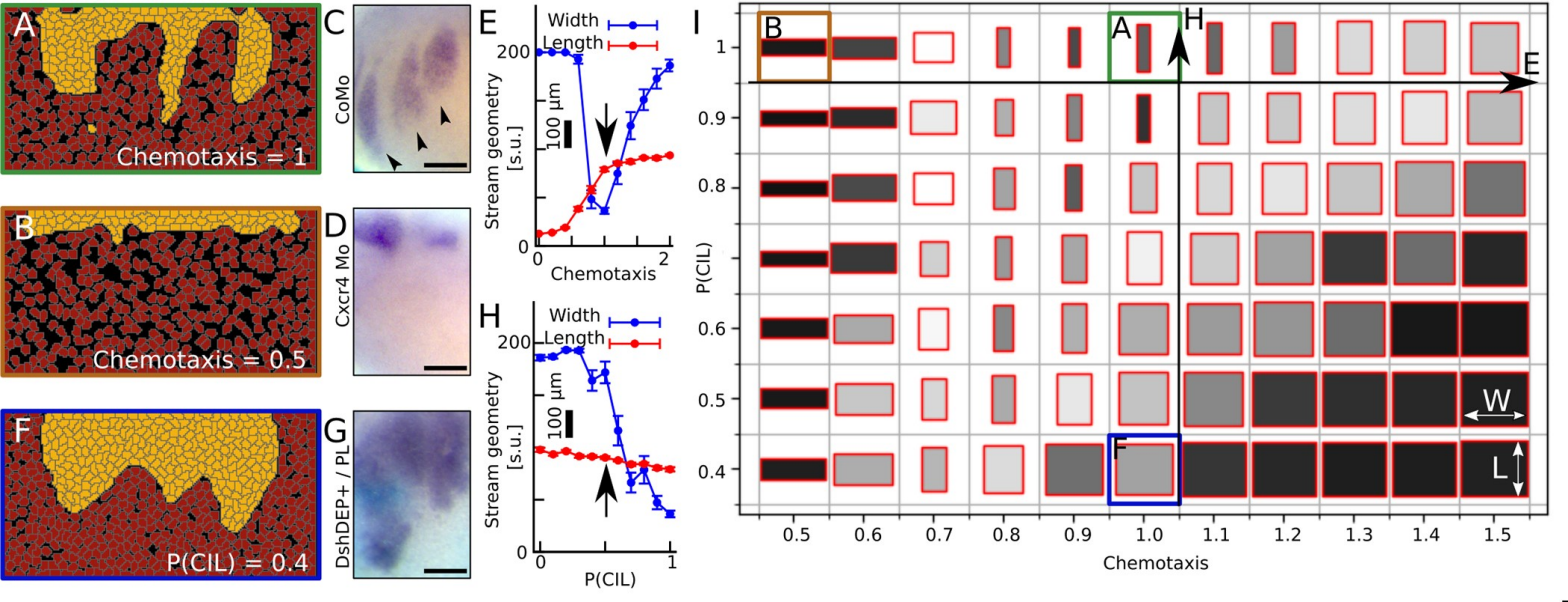
Attraction at a distance and repulsion at a shorter range is required for neural crest stream formation. (A-B) Control (A) and inhibition of Sdf1 chemotaxis (B) in simulations. Representative cell configurations at the end of simulations (t = 3600MCS). (C-D) ISH of post-migratory stage embryo injected with control-Mo (CoMo, C) showing normal NC migration and CxcR4-Mo (D) showing lack of NC migration. (E) Width and length of streams measured from the density probability functions of simulations with different chemotaxis parameters. Arrow indicates default value. (F) Inhibition of CIL in placodal cells in simulations. Configurations of cells at the end of simulation (t = 3600MCS). (G) NC shown by ISH in wild-type embryos with placodes grafted from DshDEP+ injected embryos. (H) Width and length of simulated streams as in (E), corresponding to (F) and (A). Arrow indicates default value. (I) Morphological map of simulated NC streams at various chemotaxis and placodal CIL parameters. Box width and length corresponds to width and length of streams. Box shading from light to dark corresponds decreasing relative standard error of width and length measures. Letters refer to related panels. Error bars on E and H: SEM. N = 20 simulations for each point on E, H, and I. Scalebars: 200μm.

The effect of Sdf1 chemotaxis was explored in the model by abolishing the chemotactic effect of Sdf1 on NC cells. This led to the complete absence of streams compared to control simulations (Fig 5A and 5B) indicating that in silico stream formation highly depends on Sdf1 attraction. To confirm the in silico results, the effect of Sdf1 on NC cells was abolished in vivo by inhibiting the expression of the Sdf1 receptor CxcR4 using validated morpholino oligomers (Cxcr4-Mo) (S1 Fig) [7,22]. Embryos injected with the Mo had a severe reduction of NC migration compared with control-Mo injected embryos (Fig 5C and 5D), consistent with our in silico observations.

Simulations with different chemotaxis strength parameters show that stream width exhibits a minimum, while lengths reach a plateau (Fig 5E). At higher chemotaxis strengths increase in length is limited by the compaction of placodal cells and the limitation of the model to represent out-of-plane intercalation of the placodes. We note that at low chemotaxis parameter values this result also demonstrates that EMT in the model does not produce an artificial pressure on the NC population by adding NC cells at the dorsal side (top) of the simulation area which could otherwise force NC invasion into the placode region.

To test the requirement of short-range repulsion for stream formation, the CIL behavior was removed specifically from the placodal cells in simulations. This led to a uniform invasion of the placodal region; however, it severely compromised segmentation of the NC into distinct streams compared to control simulations (Fig 5F and 5A). This result was tested experimentally by grafting whereby wild-type host embryos received graft placodes from embryos in which CIL was inhibited by injection of a dominant negative Dsh construct (DshDEP+), that specifically inhibits in frogs the PCP Wnt signaling, required for CIL [7,17]. We found that treated embryos failed to develop well-formed NC streams compared to controls where the grafts were implanted from wild-type embryos (Fig 5G). Nevertheless, NC cells still invaded the DshDEP+ placodal region, consistently with our simulation predictions.

Next, we adjusted the probability of a CIL response in placodes after collision in simulations and measured the emergent morphologies. The extent of invasion remained mostly unaffected with increasing probability of CIL reaction as signified by the length of streams (Fig 5H). Stream width, on the other hand, increases significantly as placode CIL probability decreases and reaches the maximal width at around 30% CIL probability (Fig 5H).

Finally, we investigated the interaction of the chemotaxis and contact-repulsion mechanisms in simulations. We mapped width and length of NC streams as rectangles in a 2D space of chemotaxis and CIL response parameters (Fig 5I), where shading represents variability of the measurements (light: high uncertainty, dark: low uncertainty). The simulation results reveal that chemotaxis and CIL counteract one another, yet they are both required for stream formation.

Together these results show that the chase-and-run cellular interaction between NC and placodes is essential for the formation of streams with the chase (Sdf1 chemotaxis) being responsible for powering the invasion and the run (NC-placode repulsion) being responsible for shaping the streams.

### Emergent streams are shaped by cell-cell adhesion

**Fig 6:**
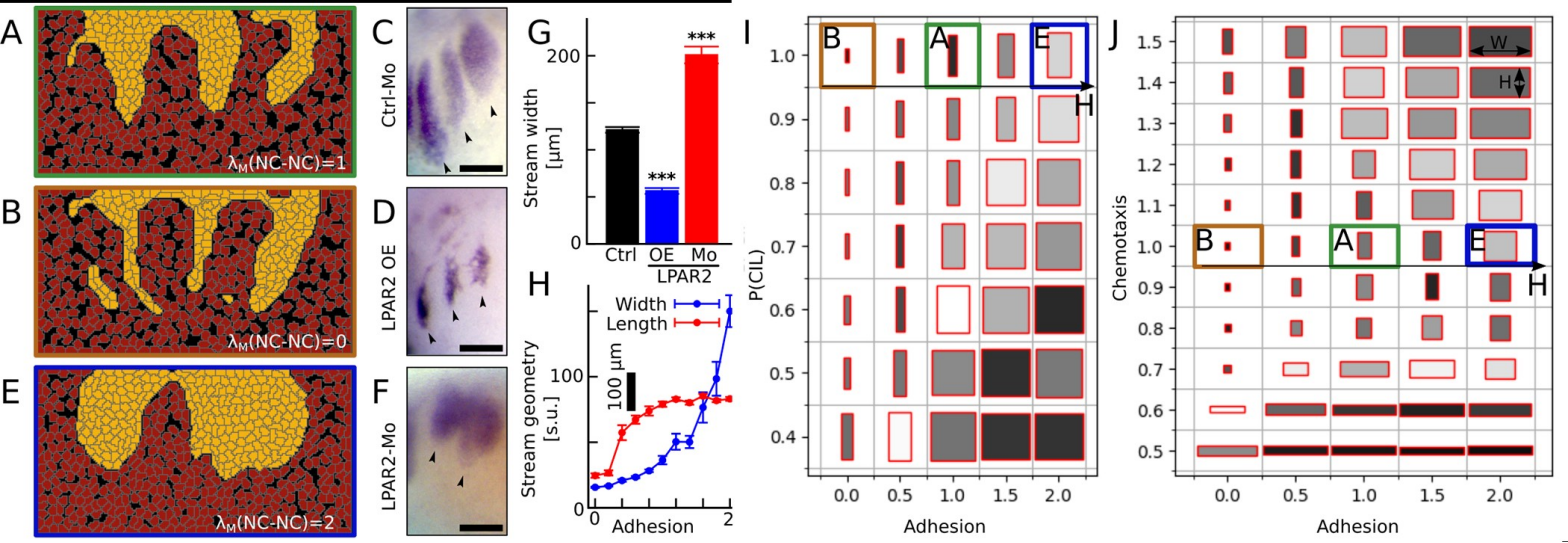
Emergent streams at different neural crest adhesion strengths. (A, B, E) Final cell configurations in simulations with normal (A), low (B), and high (E) neural crest adhesion parameters (λ_M (NC-NC)). (C, D, F) Streams revealed by ISH in control (C), LPAR2-overexpressing (D), and LPAR2-Mo injected (F) embryos. (G) Stream widths in vivo measured in control embryos and embryos injected with either LPAR2 mRNA (OE, overexpression) or LPAR2-Mo (Mo). Error bars: SEM; n(Ctrl, MO) = 30; n(OE) = 19; ***: p<0.001 Mann-Whitney test. (H) Stream width and length in silico as a function of λ_M (NC-NC). Error bars: SEM. (I, J) Morphological maps of simulated NC streams at various adhesion-placodal CIL, and adhesion-chemotaxis parameters. Box width and length corresponds to width and length of streams. Box shading from light to dark corresponds decreasing relative standard error of width and length measures. Letters refer to related panels. N = 20 simulations for each parameter set. Scalebars: 300μm.

To investigate the effect of cell-to-cell adhesion within the NC population on the emergence of streams, simulations were run with varying degrees of attachment between NC cells. Simulations with low adhesion (*λ*_*M*_(NC−NC) = 0) gave rise to thinner streams compared with control simulations (Fig 6A and 6B). As N-cadherin is the major cell adhesion molecule present in *Xenopus* cranial NC [22–25], we manipulated the adhesion in these cells by interfering with the levels of N-cadherin at the cell surface. It has been shown that in frogs the level of N-cadherin at the cell contact is controlled by N-cadherin endocytosis, which is regulated by lysophosphatidic acid receptor 2 (LPAR2)[26]. We have previously demonstrated that by changing the levels of LPAR2, adhesion strength of NC cells can be altered in frogs [26]. We decreased NC cell-cell adhesion by injecting LPAR2 mRNA, which leads to LPAR2 overexpression and gives rise to significantly thinner NC streams when compared with NC injected with a control Mo (Fig 6C, 6D and 6G) or with wild-type. This is consistent with our simulations where NC cell adhesion was weakened.

Next, we simulated an increase in cell adhesion between NC cells (*λ*_*M*_(NC−NC) = 2), which produced wide streams (Fig 6E). We increased NC cell-cell adhesion in vivo by expressing a validated LPAR2 antisense Mo (S2 Fig), which in frogs leads to strengthening of NC cell adhesions (S3 Fig)[26]. Streams in LPAR2-Mo injected embryos were significantly thicker compared with embryos expressing control Mo (Fig 6C, 6F and 6G), consistently with the simulations.

Simulations with intermediate adhesion values revealed a strong and non-linear increase of stream width with increasing adhesion (Fig 6H). Stream length, as measured from the NC density probability function $\rho (\vec{r})$, remained relatively unaffected by changes in the adhesion apart from the extreme low parameter regime, where smaller length measures result from the thinness of streams (Fig 6H).

To investigate the interplay between placodal CIL, chemotaxis, and NC-NC adhesion during stream formation, we mapped stream geometries as the function of parameters related to these behaviors (Fig 6I and 6J). Our results reveal that adhesion and placodal CIL counteracts one another: stronger adhesion requires stronger CIL for similar streams. The interaction between chemotaxis and adhesion is more complex (Fig 6J). Both strong adhesion and strong chemotaxis leads to bulk invasion, while even a strong chemotaxis can produce thinner streams when adhesion is weakened.

Taken together, our simulations and experiments show that cell-cell adhesion plays an important role in NC stream morphology.

## Discussion

Our experimental results show that migratory streams of the *Xenopus* cranial NC can form independently of their orientation with respect to the underlying mesoderm or endoderm. This questions the prevailing view that cranial NC follow a pre-pattern of environmental cues that is present prior to migration. Although a pre-pattern has been demonstrated in the path of trunk NC of mice and chick, for example by semaphorin-3F and versican [27], such demonstration is lacking in the epibranchial region prior to NC migration. At later stages of migration the cranial NC streams enter the pharyngeal arches, a series of composite structures segmenting the head [28,29]. The pattern of the arches is established through Bmp/FGF produced by the endoderm independently of the NC as shown in chick [30], which has been suggested to contribute to the NC stream pattern [31]. While the pattern of the arches explains the spacing of cranial NC streams at late migratory stages and refines the position of the NC according to the pharyngeal arches, it is unclear how early NC streams in the ectoderm would be guided by the distant endoderm. Indeed, in our ectopic graft experiments stream formation was apparently not hampered by neither the epidermis, nor the endoderm. Furthermore, cranial streams of the NC have also been observed prior to pharyngeal arch morphogenesis in fish [32,33]. The fact that NC streams and the pharyngeal arches seem to be initiated independently of one another [30] opens the question of how are they coordinated at later stages to form integrated structures.

**Fig 7:**
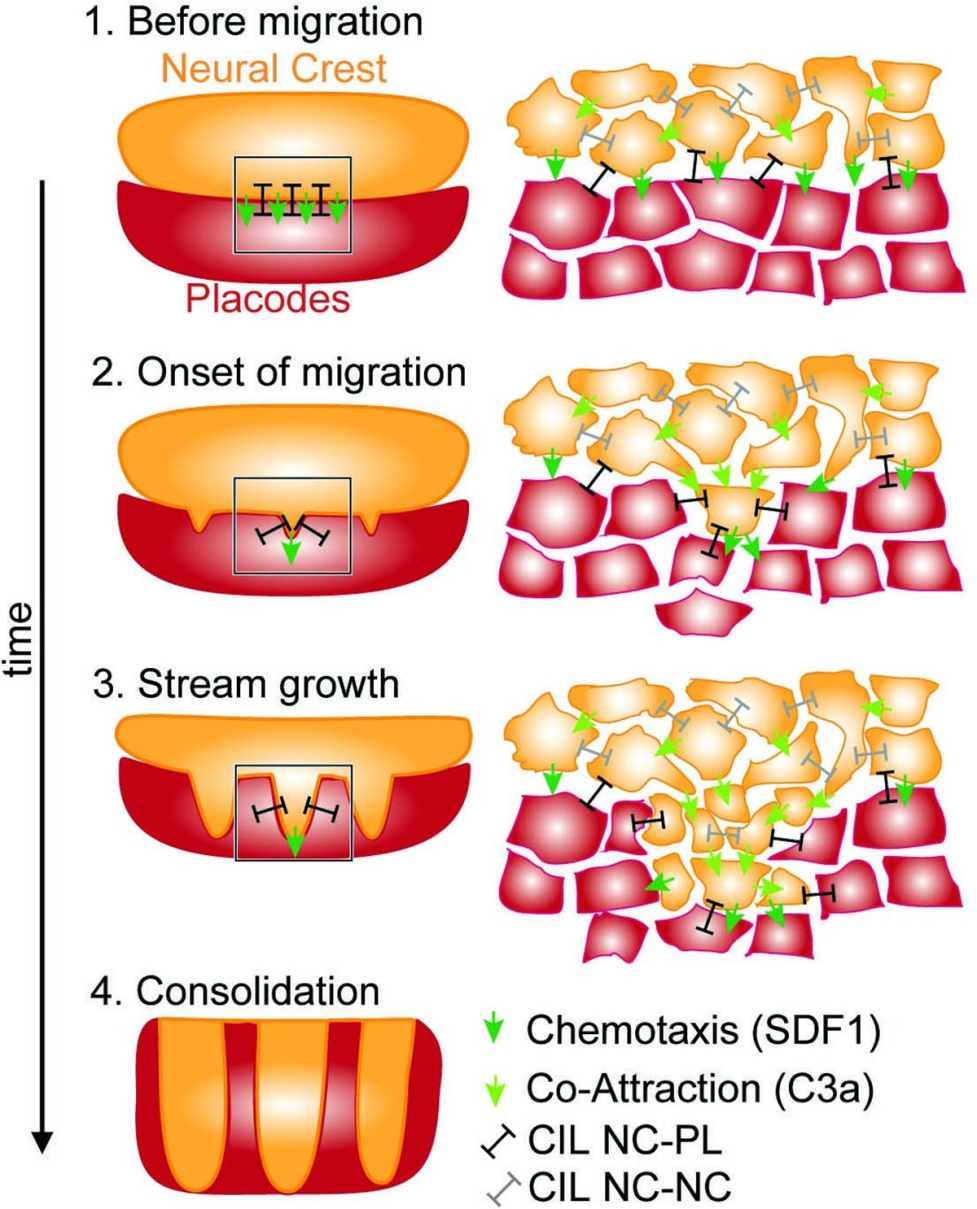
Model for emergent stream formation of the neural crest based on cellular chase and run interaction between the neighboring NC and placode populations. The gross tissue pattern (left) is determined by dynamic cell-cell interactions (right) without the need for a guiding pre-pattern to determine areas of stream growth. Consolidation of the final stream shapes at later stages are potentially facilitated by other mechanisms to ensure proper integration of the NC with other tissues.

Our findings are consistent with a model where invading NC streams emerge solely from a small set of known cellular interactions of NC and placodal cells (Fig 7). Rather than being tissue-autonomous, this model posits that patterning the NC into migratory streams depends on the presence of epibranchial placodes. This is consistent with previous findings in fish and frogs where NC streams failed to form when formation of placodes was inhibited [7] and may provide insight into why segregation of NC into distinct streams occurred together with the appearance of epibranchial placodes during evolution [34]. While our results show that this model is sufficient for forming streams, we cannot exclude the presence of a molecular pattern (inhibitory or attractive) prior to cranial NC migration. However, the effect of such a pattern on migration would be insufficient to explain our results (Fig 3).

This model can account for the observed inhibitory patterns surrounding the cranial streams (for example Fig 1C and 1D) that led to the pre-pattern hypothesis. In our model these guidance cues are expressed by the placodes and, as the placodes are compacted by the invading NC, the pattern of inhibitors emerges at the interface of the two tissues. Consistently with previous findings in chick and frogs [5,6,35], these inhibitors are required in our model for stream formation to provide the necessary repulsion between NC and placodes, but are not required to be patterned prior to migration. This is also in line with recent findings in lamprey showing that Sema3F does not guide NC segmentation directly [36]. Furthermore, in chick it has been show that the migrating NC not only require the presence of an inhibitor, but it also re-shapes the pattern of the inhibitor [37].

Our results agree with a previous work focusing on the difference between trunk and head NC migration in chicken embryos [13] which showed that the NC has a tendency to form streams. This earlier study presented a model in which invasion is driven by a global directional bias in the movement of NC cells which is hampered by a uniform extracellular matrix (ECM) distribution. When cells are allowed to digest the ECM at a faster rate, invasion in that model proceeds in a wider front [13]. This compares well with our simulations where repulsion between NC and placodes is rendered less effective, giving rise to wider NC streams (Figs 5F, 1H and 1I). Formulation of our model allows testing a wider range of parameters, leading to unstructured or even inhibited invasion. While simulations of Wynn and coworkers [13] suggest that higher NC adhesion leads to thinner invasion, our simulated and experimental results reveal an opposite trend whereby more adhesive NC tends to form wider streams (Fig 6). The different simulation results might stem from differences in model implementations and suggest experimental measurements and perturbations of cell-cell adhesions in the chick system. In support of our model, increasing cell-cell adhesion in *Xenopus* NC cells was sufficient to reduce their ability to invade narrow tunnels [26] indicating that the ability to form thin streams is inversely correlated with the strength of cell-cell adhesion. Forming thin finger-like structures from an initially wide mass of cells requires intercalation, a process that is impaired by strong cell-cell adhesion in *Xenopus* [38–40]. While our study focused on the cranial NC of *Xenopus*, investigation of the model parameters (Fig 6) suggest that this model may be suitable to describe similar pattern formation of more mesenchymal cell populations in which cell-cell adhesion is even lower. In this case, our model would predict that a higher inter-tissue attraction (chemotaxis) would be required and a lower inter-tissue repulsion (CIL) would be sufficient.

Distinction between leader and follower cells in NC streams has been reported and was explained by a difference in the gene expression levels based on single-cell sequencing in chick [15]. Although leader cells were fated to become leaders in trunk regions in fish even before migration [41], cells would be required to switch between leader and follower fates within minutes according to another study done in chick [16]. We observed individual cells at the tip of streams in our simulations which invaded the placodal population on their own. Although these cells have the exact same set of instructions (or ‘genotype’) in the model, their behavior is different from the follower cells. These leaders experience a slightly different environment than the follower cells with higher proportion of placodal neighbors, leading to a noted difference in their behavior. A similar emergent mechanism has been proposed for the selection of leading enteric NC cells in mice during the colonization of the gut where cells that get at the forefront of invasion by chance are later differentiated as leaders due to this initial advantage [42]. Our model therefore provides an alternative explanation for the emergence of persistent leader/follower positions for cranial NC migration.

Collective cell movements forming invasive finger-like structures similar to the migratory NC streams have been studied at the free edge of expanding epithelia *in vitro* [43,44] and in theory [45–47]. This phenomenon is well described by a model where invasion is enhanced at regions of high curvature [48,49]. This assumption is supported by observations in other biological systems as well [50–52] and is related to the Mullins-Sekerka instability observed typically at the solidification interface of forming crystals [53]. This instability gives a mathematical description of finger-like growth driven by the gradient of a diffusible substance. In our model, invasion of NC into the placodal region is driven by Sdf1 chemotaxis, proportional to the gradient of Sdf1, and therefore it is possible that streams are formed through such a surface instability. However, we did not observe higher chemoattractant gradients at the tips of NC streams, as would be required by the physical instability, and therefore it is unlikely that this mechanism drives stream formation in our simulations. Our system is more analogous to the pressure driven Saffman-Taylor instability [54,55], also known as viscous fingering, at the interface of two non-mixing fluids (for example honey and water). When the less viscous fluid (water) is pressed into the more viscous one (honey), the invading fluid forms finger-like protrusions with a characteristic width (*w*). In the simplest case of Newtonian fluids, *w* is determined by the surface tension between the fluids (*γ*), the velocity of invasion (*V*), the difference in fluid viscosities (*μ*), and the height of confinement in 2D (*b*) as $w∼b\sqrt{\gamma /(\mu V)}$. Measurements of tissue viscosity and surface tension in various tissues of *Xenopus* embryos estimate that *γ*/*μ*~1.8*μm*/min [56]. Considering that NC streams in *Xenopus* are relatively flat, *b*~30−50*μm* [57], and *V*~1.6*μm*/min [6], the Saffman-Taylor instability would predict stream widths in the order of *w*~50*μm*. Although this underestimates the measured 120*μm* stream width (Fig 6G), a better estimate would require measurements of *γ* and the difference in *μ* specifically for the NC and placode tissues at these stages of development. Analogy between this physical phenomenon and the appearance of NC stream pattern has previously been suggested [58] and is consistent with recent observations of changes in NC tissue tension during EMT [59]. However, a thorough investigation of the analogy is lacking and the underlying mechanisms may well be different in these seemingly similar phenomena. Formation of NC streams must be robust to accommodate unavoidable variations in embryos without affecting NC migration, therefore the NC system has to be stabilized by other mechanisms. This is partially achieved in our model by the accumulation of inhibitors (Fig 2N). However, our model focuses on the early stages of migration, on the establishment of initial streams. It is very likely that this model works together with other mechanisms, especially at later stages of migration, to refine the initial stream pattern and ensure each stream reliably reproduces its stereotypic shape in the precise location. One such important mechanism might be an interaction with the confining pharyngeal arches to achieve perfect alignment with the NC. Our model provides a numerical description of the NC system based on well-established molecular interactions between cells at a tissue interface. How parameters of our model relate to the physical parameters of the viscous finger instability is an intriguing question to be investigated in the future.

We believe that our results are applicable to all systems where two cell populations exhibit a chase-and-run behavior and are not otherwise constrained by their environment. In neural crest systems such conditions have been described in the cranial regions of *Xenopus* and zebrafish species [17]. Note that in the trunk neural crest regions the neural crest are restricted to migrate in pre-determined pathways [27,60] that are potentially present before the migration of the neural crest. Therefore, our model would not apply in this situation. Whether neural crest of other species exhibit a chase-and-run-behavior remains to be investigated. A mechanism similar to the chase-and-run has been proposed to drive the patterning of initially mixed populations of motile agents [61]. While such theoretical approaches allow a more complete understanding of the model behavior, simplicity often confounds their applicability and experimental testability.

In summary, we uncovered a general mechanism of collective invasion based on the stream formation of neural crest during early development. Our model generally predicts such patterns at the interface of tissues that exhibit long-range attraction and short-range repulsion. These cellular interactions may be present at the tumor-stroma interface [62] where they could enhance metastasis by facilitating collective invasion and may play a role in other developmental processes.

## Materials and methods

### Embryology

Embryos were obtained as previously described [6]. Briefly, *Xenopus laevis* females were injected with hCG (500U) for stimulating ovulation. Eggs were fertilized in vitro using sperm macerated in 1×MMR (100 mM NaCl, 2mM KCl, 1mM MgSO_4_, 2mM CaCl_2_, 5 mM Hepes, 100 *μ*M disodium-EDTA pH 7.6). Embryos were dejellied using 2% L-cysteine solution. When injected or for graft experiments, embryos were left to recover in normal amphibian medium (NAM) 3/8 and then transferred and maintained in NAM 1/10 [63]. NC and placode grafts were performed as previously described [7]; briefly, at mid-neurula stages (16–18 N&F) the epidermis covering the neural folds was peeled off and a band of deep ectoderm cells located in the lateral aspect of the neural fold containing prospective NC and the preplacodal region was dissected and grafted into host embryos. In *Xenopus* embryos NC and placodes are originated from the deep layer of the ectoderm and can be easily recognized by their location within the embryo and by the aspect of their cells, as it is remarkably different to all other surrounding tissues (neural plate, mesoderm, epidermis). Host NC and placode were removed before grafting and the grafted tissue was maintained in place with coverslips for several hours.

Whole-mount in situ hybridization was performed as described previously [64]. The following probes were used: Twist [65], Snail2 [66], Semaphorin-3F [21], Versican [67], Six1 [68].

### Antisense morpholino injections

Although Cxcr4 and LPAR2 Mo were characterized in previous publications [22,26] we proceed to analyze the specificity of these treatments in the context of the phenotypes described here. Inhibition of NC migration by Cxcr4 or LPAR2 Mos was efficiently rescued by co-injection of Cxcr4 or LPAR2 mRNA, respectively (S1 and S2 Figs). The mRNA used for the rescue do not bind the Mo sequence [22,26]. In addition, although we had previously shown that LPAR2 Mo affect cell-cell adhesion, we proceed to perform an additional control here. As N-cadherin is one of the major adhesion molecules expressed by *Xenopus* migrating neural crest cells [12, 69] we showed (S3 Fig) that the effect of LPAR2 Mo is completely rescued by expressing a dominant negative of N-cadherin [n-cad*Δ*] [26]. This result is consistent with the conclusion that LPAR2 Mo leads to an increase in N-cadherin at the cell junction [26] and therefore to higher cell-cell adhesion.

### Imaging

Images were acquired using a stereomicroscope (MZ FLI II; Leica Biosystems) fitted with a Plan 1.0×/0.125 objective and a camera (DFC420; Leica Biosystems). Data were acquired using IM50 v5 software (Leica Biosystems).

### Statistical analysis

Experiments were repeated at least three times independently on different weeks using different females and males for fertilization to generate embryos. Simulations were repeated 20 times using different random seeds. Data are represented as mean, spread is represented by SEM. For testing significance, the non-parametric Mann-Whitney u-test was used.

### Quantifications

Stream width in experiments was measured using ImageJ line tool on calibrated ISH images. Widths for each stream were measured at five different positions and then averaged. Number of streams was assessed manually.

### Cell-based model of neural crest—Placode interactions: Model definition

The computational model is based on our previous NC migration model [6], a cellular Potts model (CPM) [70] implemented in the Tissue Simulation Toolkit [71]. Cells in the model are represented on a 2D rectangular lattice with an integer value $\sigma (\vec{r})$ at each of the *N* lattice sites. This number identifies the cell occupying the site ($\sigma (\vec{r})>0$) and cell-free areas are denoted by $\sigma (\vec{r})=0$.

Concentrations of diffusible molecules, $c_{i}(\vec{r})$ for molecule *i* at position $\vec{r}$, are represented on the same rectangular lattice with extensions of 100 sites in all four directions to reduce boundary effects in the diffusion fields. At every time step *t* the concentrations are iterated over the extended lattice to simulate their diffusion, decay, uptake, and secretion as:

$$\frac{\partial c_{i}(t,\vec{r})}{\partial t}=D_{i}\nabla c_{i}(t,\vec{r})-\delta_{i}c_{i}(t,\vec{r})+S_{i}(\sigma (\vec{r})).$$

Here *D*_*i*_ and *δ*_*i*_ are the diffusion and decay parameters of substance *i* and $S_{i}(\sigma (\vec{r}))$ is the secretion or uptake rate of cell $\sigma (\vec{r})$ with regards to substance *i*.

Cell movement is implemented in the model as a series of attempts to overwrite a randomly chosen $\sigma (\vec{r})$ by either $\sigma (\vec{r}\prime )$ from a randomly selected neighbor $\vec{r}\prime$, or by 0 in 10% of the cases. A Monte Carlo time step (MCS) is considered to be *N* such attempts and is the unit of time in the simulations. The attempt to overwrite $\sigma (\vec{r})$ with the value *x* is accepted with a probability $p(x\to \sigma (\vec{r}))=min\{1,exp(W(x\to \sigma (\vec{r}))-\Delta H(x\to \sigma (\vec{r})))\}$. Cellular behavior therefore is determined by functions *W* and *ΔH*. The latter is the change in a function *H* as a result of the overwrite attempt which is defined over a cell configuration *ξ* as

$$H(\xi )=\sum_{i}\lambda_{V}(i)[V(i)-V_{T}(i){]}^{2}+\sum_{<x,y>}J_{x,y}[1-\Delta (x-y)].$$

The summation in the first term is taken over all cells *i*, *λ*_*V*_(*i*) is a model parameter setting the compressibility of cell *i*, *V*(*i*) and *V*_*T*_(*i*) are the area and the target area of cell *i*. This term ensures that cells maintain a relatively constant area. The second term describes surface tension-like cell-cell adhesion [70] with parameter *J*_*x*,*y*_ describing adhesion between cells *x* and *y*; *Δ*(*x* − *y*) = 1 only if *x* = *y*; the summation is taken over all possible neighboring lattice sites *x* and *y*.

Chemotaxis, persistent cell adhesions, and polarized cell movement is described through function *W* as:

$$\begin{array}{l} W(x\to \sigma (\vec{r}))=\sum_{s}\lambda_{s}(\sigma (\vec{r}))[c_{s}(t,\vec{r})-c_{s}(t,\vec{r}\prime )]\cdot \\ \;\Delta (\sigma (\vec{r}))(1-\Delta (\sigma (\vec{r}\prime )))\\ \;+\sum_{i=\sigma (\vec{r}),\sigma (\vec{r}\prime )}\lambda_{M}(i)\sum_{j\in n(i)}\frac{\vec{r_{i,j}}}{\left|\vec{r_{i,j}}\right|}(|\vec{r_{i,j}}|-d_{0})\\ \;+\sum_{i=\sigma (\vec{r}),\sigma (\vec{r}\prime )}\lambda_{P}(i)\frac{\vec{\Delta r_{i}}\vec{p(i)}}{|\vec{p(i)}} \end{array}$$ (2)

Parameters *λ*_*s*_, *λ*_*M*_, and *λ*_*P*_ set the relative weight of chemotaxis with respect to substance *s*, the persistent cell adhesions, and polarized cell movement. The first term describes extension-only chemotaxis [51] where chemotaxis only has an effect on free edge extensions of cells, not at contact sites or during retraction; summation here is over diffusing molecules *s* (CoA, Sdf1, and inhibitor). $\vec{r_{\left(i,j\right)}}$ in the second term is the vectorial distance of cells *i* and *j*, *d*_0_ is the equilibrium distance of persistently adhered cells ($d_{0}=2\sqrt{V_{T}/\pi }$), and *n*(*i*) are the persistently adhered neighbors of cell *i*. As described previously [6,72] cells in contact are connected with a 10% probability in each MCS and are disconnected once cells lose contact and otherwise with a probability of $(|\vec{r_{i,j}}|-d_{0})/100$. In the final term in Eq 2, $\vec{\Delta r_{i}}$ is the displacement of cell *i* in the last MCS and $\vec{p_{i}}$ is the polarization vector of cell *i*.

Each cell in the model possesses a polarization vector $\vec{p_{i}}$ which is updated in every MCS as $\vec{p_{i}(t+1)}=(1-\delta_{P}(i))\vec{p_{i}(t)}+\vec{\Delta r_{i}(t)}$. The value of the decay parameter *δ*_*P*_(*i*) sets the motion persistence of cells and depends on the cell type (NC or placode) and whether the cell is in contact with other cells or not as in [6].

CIL behavior is modelled as follows. When two cells undergoing CIL get in contact, each of the cells can independently switch to CIL state with a probability *p*(CIL). When a cell is in CIL state, its polarization vector is updated as $\vec{p_{i}(t+1)}=(1-\delta_{P}(i))\vec{p_{i}(t)}+\vec{\Delta r_{i}(t)}-\lambda_{CIL}(\sum_{j}\vec{r_{i,j}(t)})/|\sum_{j}\vec{r_{i,j}(t)}|$, where parameter *λ*_CIL_ sets the strength of the CIL bias, the summation covers all contacting neighbors of *i*.

### Model parameters and initial conditions

We model three diffusing substances: A (for C3a), S (for Sdf1), and I (for inhibitor) (Fig 2B, 2D and 2E). A is secreted by NC [11], while S and I are secreted by placodes [7]; in addition, S is taken up by NC cells. The parameters used for the main simulations are listed in Table 2.

Cells were initialized as 5 × 5 patches on a 180 × 95 lattice with 2 rows of NC cells and 13 rows of placodal cells (Fig 2F) and all concentrations were set to zero. As described in the main text, cell-free space is monitored at the top edge of the simulation area and whenever a region of a cell width and at least half a cell height is detected, a new NC cell is initiated there. Simulations are run for 3600 MCS. To allow comparison with experimental observations, we relate a distance of one lattice site to 3.5*μm* and one Monte Carlo time step to 10 seconds real time. With these relations, the simulated cell diameter is approximately 17*μm*, the average stream width emerging in the main simulations is 120*μm*, the total simulation time (3600MCS) is 10h, and the average simulated cell speed (0.1 pixels / MCS) converts to 2.1*μm*/min. These values are in agreement with the experimentally observed ones: the diameter of a cell spread on a surface is in the range of 10 − 25*μm*; the average stream width is 120 *μm* (Fig 6H); the approximate time required for NC stream formation and migration is 6-10h; the reported NC cell speeds are in the range of 0.5–2 *μm*/min [6].

**Table 2:** Parameters and values used in the simulations. When other values are used, they are specifically noted in the manuscript.

Parameter	Value (unless otherwise noted)	Explanation
*S*_*A*_	5 [1/MCS]	Secretion parameter for co-attractant (related to C3a)
*S*_*S*_	20 [1/MCS]	Secretion parameter for NC attractant (related to Sdf1)
*S*_*l*_	0.7 [1/MCS]	Secretion parameter for inhibitor
*D*_*A*_	8 [s.u.^2^/MCS]	Diffusion parameter for co-attractant
*D*_*S*_	8 [s.u.^2^/MCS]	Diffusion parameter for NC attractant
*D*_*I*_	0,005 [s.u.^2^ /MCS]	Diffusion parameter for inhibitor
*δ*_*A*_	0.006 [1/MCS]	Decay parameter for co-attractant
*δ*_*S*_	0.006 [1/MCS]	Decay parameter for NC attractant
*δ*_*I*_	0.0006 [1/MCS]	Decay parameter for inhibitor
*λ*_*A*_	1	Attraction strength parameter for co-attractant
$\lambda_{S}^{*}$	150 = 150 * *λ*_*S*_ = 1	Attraction strength parameter for NC attractant
*λ*_*I*_	-100	Attraction strength parameter for inhibitor
*λ*_*PNC*_	6	Self-propulsion strength parameter for NC
*δ*_*PNC*_(free)	0.3	Self-propulsion polarity decay parameter for NC without contact
*δ*_*PNC*_(contact)	0.08	Self-propulsion polarity decay parameter for NC with contact
*λ*_*PPL*_	12	Self-propulsion strength parameter for placode
*δ*_*PPL*_(free)	0.5	Self-propulsion polarity decay parameter for placode without contact
*δ*_*PPL*_(contact)	0.1	Self-propulsion polarity decay parameter for placode with contact
*λ*_CIL_	0.5	CIL repolarization parameter
*λ*_*M*_	5	Persistent cell-cell adhesion strength parameter
*λ*_*V*_	5	Cell incompressibility parameter
*V*_*T*_	25 [s.u.^2^]	Cell volume
*J*(NC,0)	5	Surface-tension-like adhesion between NC and cell-free areas
*J*(NC,NC)	3	Surface-tension-like adhesion between NC
*J*(placode,0)	5	Surface-tension-like adhesion between placodes and cell-free areas
*J*(placode,NC)	20	Surface-tension-like adhesion between placodes and NC
*J*(placode,placode)	10	Surface-tension-like adhesion between placodes

## Supporting information

S1 Fig Inhibition of NC migration by Cxcr4-Mo is efficiently rescued by co-injection of Cxcr4 mRNA showing specificity of the Mo treatment. (A) Lateral view of representative embryos injected with Control-Mo (CoMo), Cxcr4-Mo, and Cxcr4-Mo + Cxcr4 mRNA; scalebars: 250μm. (B) Ratio of embryos with normal NC migration in control (CoMo), inhibition (Cxcr4-Mo), and rescue (Cxcr4-Mo+mRNA) experiments. N = 4 experiments, with 50 embryos each; bars: mean, errorbars: SEM; ***: p<0.001, ns: p>0.05 (t-test).
(TIF)

S2 Fig Inhibition of NC migration by LPAR2-Mo is efficiently rescued by co-injection of LPAR2 mRNA showing specificity of the Mo treatment. (A) Lateral view of representative embryos injected with Control-Mo (CoMo), LPAR2-Mo, and LPAR2-Mo + LPAR2 mRNA; scalebars: 250μm. (B) Ratio of embryos with normal NC migration in control (CoMo), inhibition (LPAR2-Mo), and rescue (LPAR2-Mo+mRNA) experiments. N = 3 experiments, with 45 embryos each; bars: mean, errorbars: SEM; ***: p<0.001, ns: p>0.05 (t-test).
(TIF)

S3 Fig LPAR2 Mo affect cell-cell adhesion. (A) Lateral view of embryos injected with LPAR2-Mo and LPAR2-Mo + dominant negative of N-cadherin (N-Cad*Δ*); scalebars: 250μm. (B) Ratio of embryos with inhibition (LPAR2-Mo) and rescue (LPAR2-Mo+N-Cad*Δ*) experiments. N = 5 experiments, with 40 embryos each; bars: mean, errorbars: SEM. Note that effect of LPAR2 Mo is completely rescued by expressing a dominant negative of N-cadherin. As N-cadherin is the major adhesion molecule expressed by *Xenopus* migrating neural crest cells [69] these results confirm previous publications showing that LPAR2 Mo leads to an increase in N-cadherin at the cell junction [26] and therefore to higher cell-cell adhesion.
(TIF)

S1 Movie Simulation of stream formation emerging from cell-cell interactions. Frame rate shown in Monte Carlo time steps (MCS), total length 3600 MCS. Red: placodal cells, orange: NC cells, black: cell-free area.
(AVI)

S2 Movie Simulation of stream formation emerging from cell-cell interactions, showing the evolution of inhibitor concentration distribution. Color-code shows normalized concentration levels. Frame rate shown in Monte Carlo time steps (MCS), total length 3600 MCS.
(AVI)

## References

[1] Keller RE, Shook D, Skoglund P. The forces that shape embryos: physical aspects of convergent extension by cell intercalation. Phys Biol. 2008;5: 015007 10.1088/1478-3975/5/1/015007 18403829

[2] Qiao J, Sakurai H, Nigam SK. Branching morphogenesis independent of mesenchymal-epithelial contact in the developing kidney. Proc Natl Acad Sci. 1999;96: 7330–7335. 10.1073/pnas.96.13.7330 10377414 PMC22085

[3] Varner VD, Gleghorn JP, Miller E, Radisky DC, Nelson CM. Mechanically patterning the embryonic airway epithelium. Proc Natl Acad Sci. 2015;112: 9230–9235. 10.1073/pnas.1504102112 26170292 PMC4522767

[4] Szabó A, Mayor R. Mechanisms of Neural Crest Migration. Annu Rev Genet. 2018;52: 43–63. 10.1146/annurev-genet-120417-031559 30476447

[5] Gammill LS, Gonzalez C, Bronner-Fraser M. Neuropilin 2/semaphorin 3F signaling is essential for cranial neural crest migration and trigeminal ganglion condensation. Dev Neurobiol. 2007;67: 47–56. 10.1002/dneu.20326 17443771

[6] Szabó A, Melchionda M, Nastasi G, Woods ML, Campo S, Perris R, et al In vivo confinement promotes collective migration of neural crest cells. J Cell Biol. 2016;213: 543–555. 10.1083/jcb.201602083 27241911 PMC4896058

[7] Theveneau E, Steventon B, Scarpa E, Garcia S, Trepat X, Streit A, et al Chase-and-run between adjacent cell populations promotes directional collective migration. Nat Cell Biol. Nature Publishing Group; 2013;15: 763–72. 10.1038/ncb2772 23770678 PMC4910871

[8] Shiau CE, Lwigale PY, Das RM, Wilson SA, Bronner-Fraser M. Robo2-Slit1 dependent cell-cell interactions mediate assembly of the trigeminal ganglion. Nat Neurosci. 2008;11: 269–276. 10.1038/nn2051 18278043

[9] Smith A, Robinson V, Patel K, Wilkinson DG. The EphA4 and EphB1 receptor tyrosine kinases and ephrin-B2 ligand regulate targeted migration of branchial neural crest cells. Curr Biol. 1997;7: 561–570. 10.1016/S0960-9822(06)00255-7 9259557

[10] Golding JP, Trainor P, Krumlauf R, Gassmann M. Defects in pathfinding by cranial neural crest cells in mice lacking the neuregulin receptor ErbB4. Nat Cell Biol. 2000;2: 103–109. 10.1038/35000058 10655590

[11] Carmona-Fontaine C, Theveneau E, Tzekou A, Tada M, Woods ML, Page KM, et al Complement fragment C3a controls mutual cell attraction during collective cell migration. Dev Cell. Elsevier Inc.; 2011;21: 1026–37. 10.1016/j.devcel.2011.10.012 22118769 PMC3272547

[12] McLennan R, Dyson L, Prather KW, Morrison J a, Baker RE, Maini PK, et al Multiscale mechanisms of cell migration during development: theory and experiment. Development. 2012;139: 2935–44. 10.1242/dev.081471 22764050 PMC3403103

[13] Wynn ML, Rupp P, Trainor PA, Schnell S, Kulesa PM. Follow-the-leader cell migration requires biased cell-cell contact and local microenvironmental signals. Phys Biol. 2013;10: 035003 10.1088/1478-3975/10/3/035003 23735560 PMC3756809

[14] Woods ML, Carmona-Fontaine C, Barnes CP, Couzin ID, Mayor R, Page KM. Directional Collective Cell Migration Emerges as a Property of Cell Interactions. Simpson MJ, editor. PLoS One. 2014;9: e104969 10.1371/journal.pone.0104969 25181349 PMC4152153

[15] McLennan R, Schumacher LJ, Morrison J a., Teddy JM, Ridenour D a, o a. C, et al Neural crest migration is driven by a few trailblazer cells with a unique molecular signature narrowly confined to the invasive front. Development. 2015; 1–12. 10.1242/dev.119727 25977364

[16] McLennan R, Schumacher LJ, Morrison JA, Teddy JM, Ridenour DA, Box AC, et al VEGF signals induce trailblazer cell identity that drives neural crest migration. Dev Biol. Elsevier; 2015;407: 12–25. 10.1016/j.ydbio.2015.08.011 26278036

[17] Carmona-Fontaine C, Matthews HK, Kuriyama S, Moreno M, Dunn GA, Parsons M, et al Contact inhibition of locomotion in vivo controls neural crest directional migration. Nature. 2008;456: 957–61. 10.1038/nature07441 19078960 PMC2635562

[18] Roycroft A, Mayor R. Molecular basis of contact inhibition of locomotion. Cell Mol Life Sci. Springer Basel; 2015;25: 373–375. 10.1007/s00018-015-2090-0 PMC4761371 26585026

[19] Nieuwkoop P, Faber J. Normal table of Xenopus laevis Amsterdam: North Holland Publishing Co; 1967.

[20] Streit A. Early development of the cranial sensory nervous system: from a common field to individual placodes. Dev Biol. 2004;276: 1–15. 10.1016/j.ydbio.2004.08.037 15531360

[21] Koestner U, Shnitsar I, Linnemannstöns K, Hufton AL, Borchers A. Semaphorin and neuropilin expression during early morphogenesis of Xenopus laevis. Dev Dyn. 2008;237: 3853–3863. 10.1002/dvdy.21785 18985750

[22] Theveneau E, Marchant L, Kuriyama S, Gull M, Moepps B, Parsons M, et al Collective chemotaxis requires contact-dependent cell polarity. Dev Cell. Elsevier Ltd; 2010;19: 39–53. 10.1016/j.devcel.2010.06.012 20643349 PMC2913244

[23] Bahm I, Barriga EH, Frolov A, Theveneau E, Frankel P, Mayor R. PDGF controls contact inhibition of locomotion by regulating N-cadherin during neural crest migration. Development. 2017;144: 2456–2468. 10.1242/dev.147926 28526750 PMC5536867

[24] Huang C, Kratzer M-C, Wedlich D, Kashef J. E-cadherin is required for cranial neural crest migration in Xenopus laevis. Dev Biol. Elsevier; 2016;411: 159–171. 10.1016/j.ydbio.2016.02.007 26879760

[25] Scarpa E, Szabó A, Bibonne A, Theveneau E, Parsons M, Mayor R. Cadherin Switch during EMT in Neural Crest Cells Leads to Contact Inhibition of Locomotion via Repolarization of Forces. Dev Cell. 2015;34: 421–434. 10.1016/j.devcel.2015.06.012 26235046 PMC4552721

[26] Kuriyama S, Theveneau E, Benedetto A, Parsons M, Tanaka M, Charras GT, et al In vivo collective cell migration requires an LPAR2-dependent increase in tissue fluidity. J Cell Biol. 2014;206: 113–27. 10.1083/jcb.201402093 25002680 PMC4085712

[27] Gammill LS, Gonzalez C, Gu C, Bronner-Fraser M. Guidance of trunk neural crest migration requires neuropilin 2/semaphorin 3F signaling. Development. 2006;133: 99–106. 10.1242/dev.02187 16319111

[28] Shone V, Graham A. Endodermal/ectodermal interfaces during pharyngeal segmentation in vertebrates. J Anat. 2014;225: 479–491. 10.1111/joa.12234 25201771 PMC4292749

[29] Graham A, Smith A. Patterning the pharyngeal arches. BioEssays. John Wiley & Sons, Inc.; 2000;23: 54–61. 10.1002/1521-1878(200101)23:1<54::AID-BIES1007>3.0.CO;2-5 11135309

[30] Veitch E, Begbie J, Schilling TF, Smith MM, Graham A. Pharyngeal arch patterning in the absence of neural crest. Curr Biol. Cell Press; 1999;9: 1481–1484. 10.1016/S0960-9822(00)80118-9 10607595

[31] Cerny R, Lwigale P, Ericsson R, Meulemans D, Epperlein H-H, Bronner-Fraser M. Developmental origins and evolution of jaws: new interpretation of “maxillary” and “mandibular.” Dev Biol. Academic Press; 2004;276: 225–236. 10.1016/j.ydbio.2004.08.046 15531376

[32] Schilling TF, Kimmel CB. Segment and cell type lineage restrictions during pharyngeal arch development in the zebrafish embryo. Development. 1994;120: 483–94. Available: http://www.ncbi.nlm.nih.gov/pubmed/8162849 8162849 10.1242/dev.120.3.483

[33] Kimmel CB, Miller CT, Keynes RJ. Neural crest patterning and the evolution of the jaw. J Anat. Blackwell Science Ltd; 2001;199: 105–120. 10.1046/j.1469-7580.2001.19910105.x 11523812 PMC1594948

[34] Graham A. The development and evolution of the pharyngeal arches. J Anat. Blackwell Science Ltd; 2001;199: 133–141. 10.1046/j.1469-7580.2001.19910133.x 11523815 PMC1594982

[35] Perissinotto D, Iacopetti P, Bellina I, Doliana R, Colombatti A, Pettway Z, et al Avian neural crest cell migration is diversely regulated by the two major hyaluronan-binding proteoglycans PG-M/versican and aggrecan. Development. 2000;127: 2823–42. Available: http://www.ncbi.nlm.nih.gov/pubmed/10851128 10851128 10.1242/dev.127.13.2823

[36] York JR, Yuan T, Lakiza O, McCauley DW. An ancestral role for Semaphorin3F-Neuropilin signaling in patterning neural crest within the new vertebrate head. Development. 2018;145: dev164780 10.1242/dev.164780 29980564

[37] McLennan R, Bailey CM, Schumacher LJ, Teddy JM, Morrison JA, Kasemeier-Kulesa JC, et al DAN (NBL1) promotes collective neural crest migration by restraining uncontrolled invasion. J Cell Biol. 2017;216: 3339–3354. 10.1083/jcb.201612169 28811280 PMC5626539

[38] Lee C-H, Gumbiner BM. Disruption of Gastrulation Movements in Xenopus by a Dominant-Negative Mutant for C-cadherin. Dev Biol. 1995;171: 363–373. 10.1006/dbio.1995.1288 7556920

[39] Brieher WM. Regulation of C-cadherin function during activin induced morphogenesis of Xenopus animal caps. J Cell Biol. 1994;126: 519–527. 10.1083/jcb.126.2.519 8034750 PMC2200019

[40] Zhong Y, Brieher WM, Gumbiner BM. Analysis of C-cadherin Regulation during Tissue Morphogenesis with an Activating Antibody. J Cell Biol. 1999;144: 351–359. 10.1083/jcb.144.2.351 9922460 PMC2132887

[41] Richardson J, Gauert A, Briones Montecinos L, Fanlo L, Alhashem ZM, Assar R, et al Leader Cells Define Directionality of Trunk, but Not Cranial, Neural Crest Cell Migration. Cell Rep. 2016;15: 2076–2088. 10.1016/j.celrep.2016.04.067 27210753 PMC4893160

[42] Cheeseman BL, Zhang D, Binder BJ, Newgreen DF, Landman KA. Cell lineage tracing in the developing enteric nervous system: superstars revealed by experiment and simulation. J R Soc Interface. 2014;11: 20130815–20130815. 10.1098/rsif.2013.0815 24501272 PMC3928926

[43] Reffay M, Parrini MC, Cochet-Escartin O, Ladoux B, Buguin A, Coscoy S, et al Interplay of RhoA and mechanical forces in collective cell migration driven by leader cells. Nat Cell Biol. 2014; 10.1038/ncb2917 24561621

[44] Petitjean L, Reffay M, Grasland-Mongrain E, Poujade M, Ladoux B, Buguin A, et al Velocity fields in a collectively migrating epithelium. Biophys J. Biophysical Society; 2010;98: 1790–1800. 10.1016/j.bpj.2010.01.030 20441742 PMC2862185

[45] Kessler DA, Levine H. Microscopic Selection of Fluid Fingering Patterns. Phys Rev Lett. 2001;86: 4532–4535. 10.1103/PhysRevLett.86.4532 11384276

[46] Basan M, Elgeti J, Hannezo E, Rappel W-J, Levine H. Alignment of cellular motility forces with tissue flow as a mechanism for efficient wound healing. Proc Natl Acad Sci. 2013;110: 2452–2459. 10.1073/pnas.1219937110 23345440 PMC3574962

[47] Zimmermann J, Basan M, Levine H. An instability at the edge of a tissue of collectively migrating cells can lead to finger formation during wound healing. Eur Phys J Spec Top. 2014;223: 1259–1264. 10.1140/epjst/e2014-02189-7

[48] Mark S, Shlomovitz R, Gov NS, Poujade M, Grasland-Mongrain E, Silberzan P. Physical Model of the Dynamic Instability in an Expanding Cell Culture. Biophys J. Elsevier; 2010;98: 361–370. 10.1016/j.bpj.2009.10.022 20141748 PMC2814206

[49] Tarle V, Ravasio A, Hakim V, Gov NS. Modeling the finger instability in an expanding cell monolayer. Integr Biol. Royal Society of Chemistry; 2015;7: 1218–1227. 10.1039/C5IB00092K 26099063

[50] Nelson CM, Vanduijn MM, Inman JL, Fletcher D a, Bissell MJ. Tissue geometry determines sites of mammary branching morphogenesis in organotypic cultures. Science (80-). 2006;314: 298–300. 10.1126/science.1131000 17038622 PMC2933179

[51] Merks RMH, Perryn ED, Shirinifard A, Glazier JA. Contact-Inhibited Chemotaxis in De Novo and Sprouting Blood-Vessel Growth. Bourne PE, editor. PLoS Comput Biol. Public Library of Science; 2008;4: 16 Available: http://arxiv.org/abs/q-bio/0505033 10.1371/journal.pcbi.1000163 PMC2528254 18802455

[52] Czirók A. Endothelial cell motility, coordination and pattern formation during vasculogenesis. Wiley Interdiscip Rev Syst Biol Med. 2013;5: 587–602. 10.1002/wsbm.1233 23857825 PMC3737767

[53] Mullins W, Sekerka R. Stability of a planar interface during solidification of a dilute binary alloy. J Appl Phys. 1964;444 10.1063/1.1713333

[54] Saffman PG, Taylor G. The Penetration of a Fluid into a Porous Medium or Hele-Shaw Cell Containing a More Viscous Liquid. Proc R Soc A Math Phys Eng Sci. 1958;245: 312–329. 10.1098/rspa.1958.0085

[55] Couder Y. Viscous Fingering as an Archetype for Growth Patterns. Perspectives in Fluid Dymanics. 2003 p. 53.

[56] David R, Luu O, Damm EW, Wen JWH, Nagel M, Winklbauer R. Tissue cohesion and the mechanics of cell rearrangement. Development. 2014;141: 3672–3682. 10.1242/dev.104315 25249459

[57] Barriga EH, Franze K, Charras G, Mayor R. Tissue stiffening coordinates morphogenesis by triggering collective cell migration in vivo. Nature. 2018;554: 523–527. 10.1038/nature25742 29443958 PMC6013044

[58] Newman SA, Comper WD. “Generic” physical mechanisms of morphogenesis and pattern formation. Development. 1990;110: 1–18. Available: http://www.ncbi.nlm.nih.gov/pubmed/2081452 2081452 10.1242/dev.110.1.1

[59] Blaue C, Kashef J, Franz CM. Cadherin-11 promotes neural crest cell spreading by reducing intracellular tension—mapping adhesion and mechanics in neural crest explants by atomic force microscopy. Semin Cell Dev Biol. Elsevier Ltd; 2017; 10.1016/j.semcdb.2017.08.058 28919310

[60] Dutt S, Kléber M, Matasci M, Sommer L, Zimmermann DR. Versican V0 and V1 guide migratory neural crest cells. J Biol Chem. 2006;281: 12123–31. 10.1074/jbc.M510834200 16510447

[61] Dini S, Binder BJ, Green JEF. Understanding interactions between populations: Individual based modelling and quantification using pair correlation functions. J Theor Biol. Academic Press; 2018;439: 50–64. 10.1016/j.jtbi.2017.11.014 29197512

[62] Labernadie A, Kato T, Brugués A, Serra-Picamal X, Derzsi S, Arwert E, et al A mechanically active heterotypic E-cadherin/N-cadherin adhesion enables fibroblasts to drive cancer cell invasion. Nat Cell Biol. 2017;19: 224–237. 10.1038/ncb3478 28218910 PMC5831988

[63] Beck CW, Slack JM. A developmental pathway controlling outgrowth of the Xenopus tail bud. Development. 1999;126: 1611–1620. 10079224 10.1242/dev.126.8.1611

[64] Harland RM. Xenopus laevis: Practical Uses in Cell and Molecular Biology [Internet]. Methods in cell biology. Elsevier; 1991 Available: http://www.ncbi.nlm.nih.gov/pubmed/1811161 1811129

[65] Hopwood ND, Pluck A, Gurdon J. A Xenopus mRNA related to Drosophila twist is expressed in response to induction in the mesoderm and the neural crest. Cell. 1989;59: 893–903. 10.1016/0092-8674(89)90612-0 2590945

[66] Mayor R, Morgan R, Sargent MG. Induction of the prospective neural crest of Xenopus. Development. 1995;121: 767–777. 7720581 10.1242/dev.121.3.767

[67] Casini P, Ori M, Avenoso A, D’Ascola A, Traina P, Mattina W, et al Identification and gene expression of versican during early development of Xenopus. Int J Dev Biol. 2008;52: 993–8. 10.1387/ijdb.082582pc 18956330

[68] Ghanbari H, Seo HC, Fjose A, Brändli a W. Molecular cloning and embryonic expression of Xenopus Six homeobox genes. Mech Dev. 2001;101: 271–277. 10.1016/S0925-4773(00)00572-4 11231090

[69] Kashef J, Kohler A, Kuriyama S, Alfandari D, Mayor R, Wedlich D. Cadherin-11 regulates protrusive activity in Xenopus cranial neural crest cells upstream of Trio and the small GTPases. Genes Dev. 2009;23: 1393–1398. 10.1101/gad.519409 19528317 PMC2701577

[70] Graner F, Glazier JA. Simulation of biological cell sorting using a two-dimensional extended Potts model. Phys Rev Lett. APS; 1992;69: 2013–2016. Available: 10.1103/PhysRevLett.69.2013 10046374

[71] Daub JT, Merks RMH. Cell-based computational modeling of vascular morphogenesis using tissue simulation toolkit. Ribatti D, editor. Methods Mol Biol. New York, NY: Springer New York; 2015;1214: 67–127. 10.1007/978-1-4939-1462-3_6 25468600

[72] Czirók A, Varga K, Méhes E, Szabó A. Collective cell streams in epithelial monolayers depend on cell adhesion. New J Phys. 2013;15: 075006 10.1088/1367-2630/15/7/075006 24363603 PMC3866308

