# Supplementary figures and images for: Neural crest streaming as an emergent property of tissue interactions during morphogenesis
Source: PLoS Comput Biol. 2019 Apr 22;15(4):e1007002. doi: 10.1371/journal.pcbi.1007002 (PMC6497294; doi:10.1371/journal.pcbi.1007002)

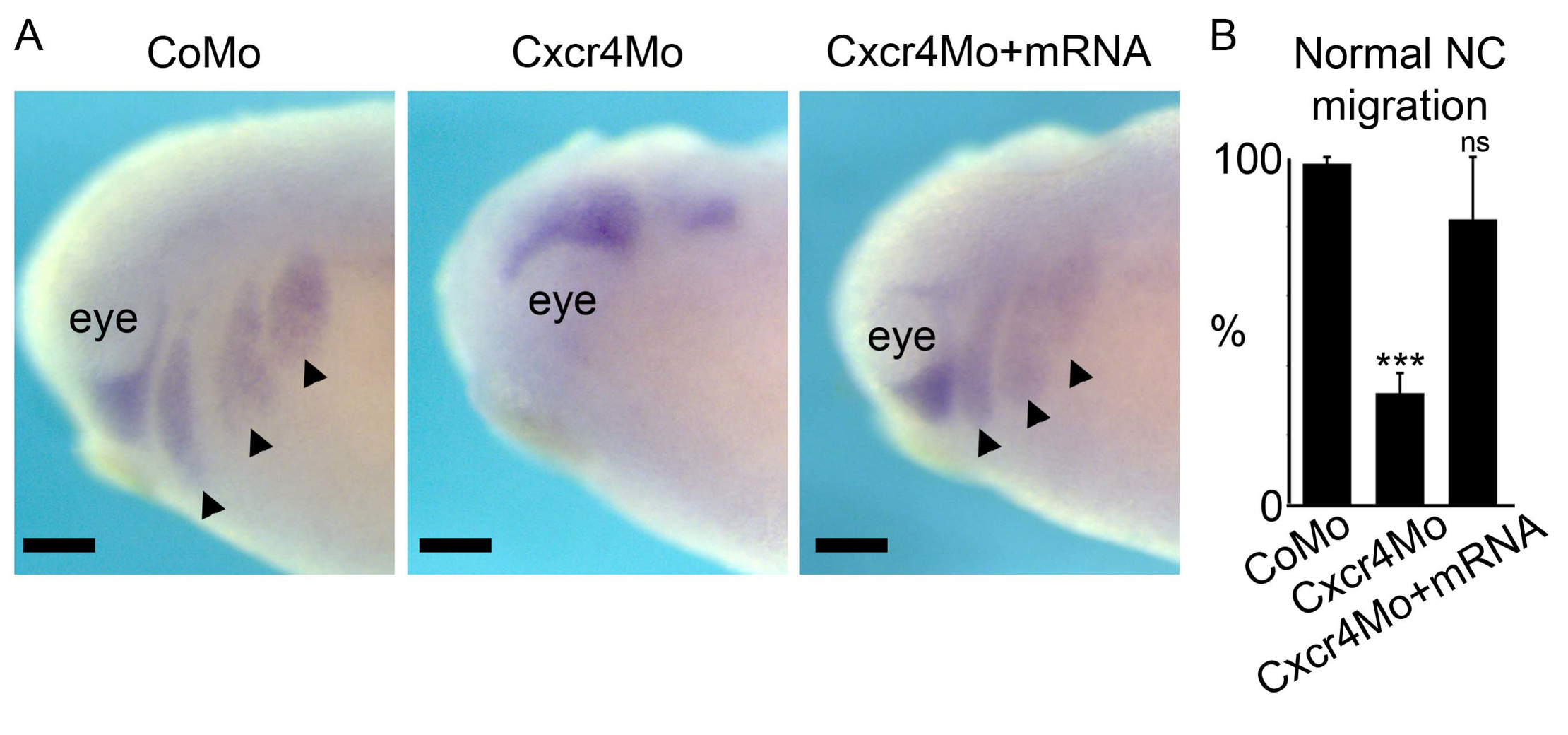

Supplement: S1 Fig — (A) Lateral view of representative embryos injected with Control-Mo (CoMo), Cxcr4-Mo, and Cxcr4-Mo + Cxcr4 mRNA; scalebars: 250μm. (B) Ratio of embryos with normal NC migration in control (CoMo), inhibition (Cxcr4-Mo), and rescue (Cxcr4-Mo+mRNA) experiments. N = 4 experiments, with 50 embryos each; bars: mean, errorbars: SEM; ***: p<0.001, ns: p>0.05 (t-test). (TIF) [file pcbi.1007002.s001.tif]

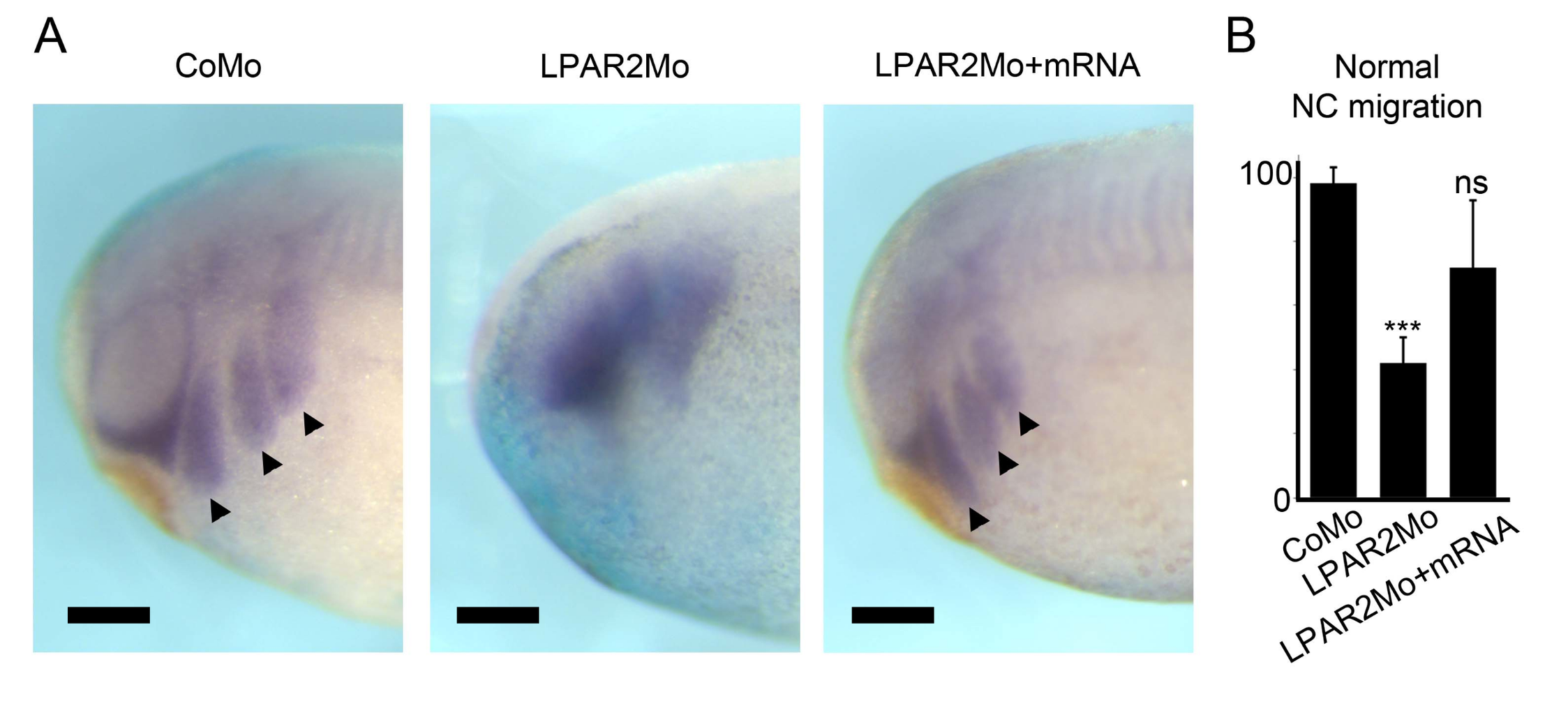

Supplement: S2 Fig — (A) Lateral view of representative embryos injected with Control-Mo (CoMo), LPAR2-Mo, and LPAR2-Mo + LPAR2 mRNA; scalebars: 250μm. (B) Ratio of embryos with normal NC migration in control (CoMo), inhibition (LPAR2-Mo), and rescue (LPAR2-Mo+mRNA) experiments. N = 3 experiments, with 45 embryos each; bars: mean, errorbars: SEM; ***: p<0.001, ns: p>0.05 (t-test). (TIF) [file pcbi.1007002.s002.tif]

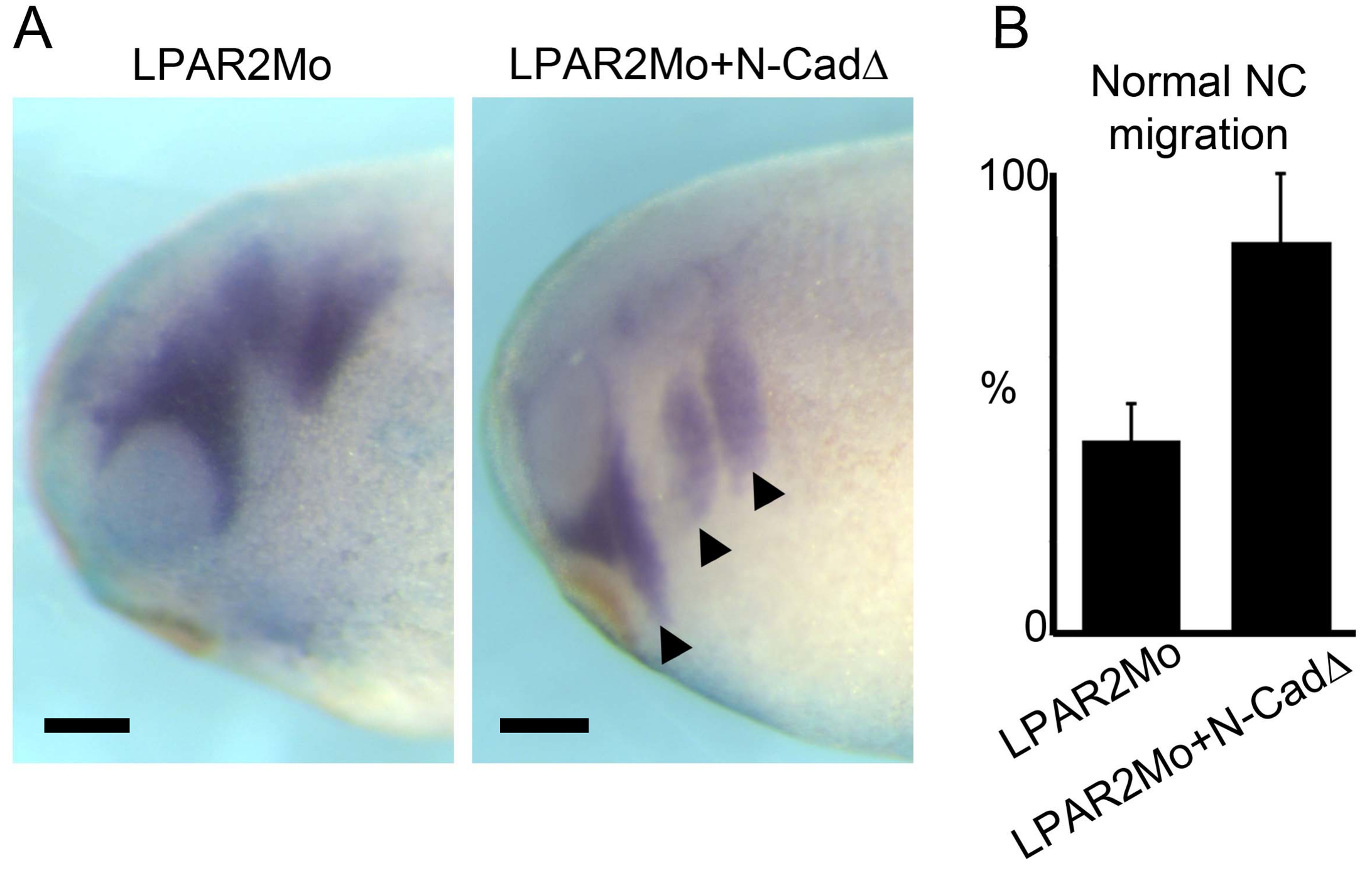

Supplement: S3 Fig — (A) Lateral view of embryos injected with LPAR2-Mo and LPAR2-Mo + dominant negative of N-cadherin (N-CadΔ); scalebars: 250μm. (B) Ratio of embryos with inhibition (LPAR2-Mo) and rescue (LPAR2-Mo+N-CadΔ) experiments. N = 5 experiments, with 40 embryos each; bars: mean, errorbars: SEM. Note that effect of LPAR2 Mo is completely rescued by expressing a dominant negative of N-cadherin. As N-cadherin is the major adhesion molecule expressed by Xenopus migrating neural crest cells [69] these results confirm previous publications showing that LPAR2 Mo leads to an increase in N-cadherin at the cell junction [26] and therefore to higher cell-cell adhesion. (TIF) [file pcbi.1007002.s003.tif]
